# Determining the molecular drivers of species-specific interferon-stimulated gene product 15 interactions with nairovirus ovarian tumor domain proteases

**DOI:** 10.1371/journal.pone.0226415

**Published:** 2019-12-23

**Authors:** John V. Dzimianski, Florine E. M. Scholte, Isabelle L. Williams, Caroline Langley, Brendan T. Freitas, Jessica R. Spengler, Éric Bergeron, Scott D. Pegan

**Affiliations:** 1 Department of Pharmaceutical and Biomedical Sciences, University of Georgia, Athens, Georgia, United States of America; 2 Division of High Consequence Pathogens and Pathology, Viral Special Pathogens Branch, Centers for Disease Control and Prevention, Atlanta, Georgia, United States of America; University of Iowa, UNITED STATES

## Abstract

Tick-borne nairoviruses (order *Bunyavirales*) encode an ovarian tumor domain protease (OTU) that suppresses the innate immune response by reversing the post-translational modification of proteins by ubiquitin (Ub) and interferon-stimulated gene product 15 (ISG15). Ub is highly conserved across eukaryotes, whereas ISG15 is only present in vertebrates and shows substantial sequence diversity. Prior attempts to address the effect of ISG15 diversity on viral protein-ISG15 interactions have focused on only a single species’ ISG15 or a limited selection of nairovirus OTUs. To gain a more complete perspective of OTU-ISG15 interactions, we biochemically assessed the relative activities of 14 diverse nairovirus OTUs for 12 species’ ISG15 and found that ISG15 activity is predominantly restricted to particular nairovirus lineages reflecting, in general, known virus-host associations. To uncover the underlying molecular factors driving OTUs affinity for ISG15, X-ray crystal structures of Kupe virus and Ganjam virus OTUs bound to sheep ISG15 were solved and compared to complexes of Crimean-Congo hemorrhagic fever virus and Erve virus OTUs bound to human and mouse ISG15, respectively. Through mutational and structural analysis seven residues in ISG15 were identified that predominantly influence ISG15 species specificity among nairovirus OTUs. Additionally, OTU residues were identified that influence ISG15 preference, suggesting the potential for viral OTUs to adapt to different host ISG15s. These findings provide a foundation to further develop research methods to trace nairovirus-host relationships and delineate the full impact of ISG15 diversity on nairovirus infection.

## Introduction

**Fig 1:**
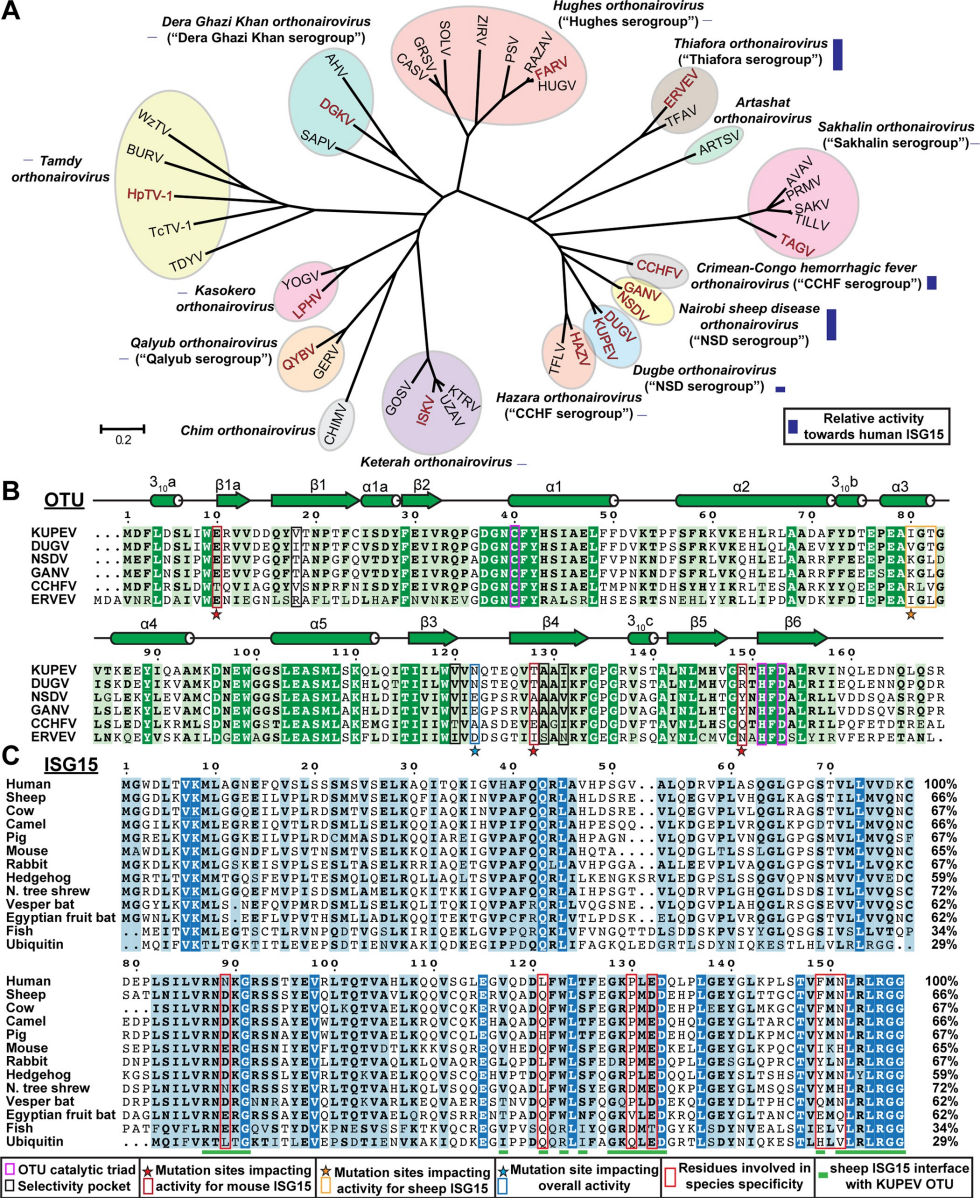
Sequence alignment of OTUs and ISG15. (A) Phylogenetic tree of Nairoviruses based on the OTU sequence, adapted from Dzimianski et al. 2019 [41]. Viruses belonging to the same species are shown by colored ovals, with serogroup classifications noted where applicable. Viruses included in this study are indicated with red text. The relative human deISGylase activity of representative OTUs within 12 of the species is shown by a purple bar based on previous studies. (B) Sequence alignment of the OTUs from KUPEV, DUGV, GANV, NSDV, CCHFV, and ERVEV. A generic secondary structure of the nairovirus OTUs is shown. Residue numbering is based on the KUPEV OTU. Residues forming the catalytic triad are boxed in magenta, while those forming the “selectivity pocket” previously identified as important for interaction with Ub are boxed in black. Mutation sites targeted to influence overall activity is indicated by a blue star/box, sites to influence sheep ISG15 activity with an orange star/orange box, and sites to influence mouse ISG15 activity with a red star/box. An expanded sequence alignment with the remaining OTUs is included in the S1 Fig. (C) Alignment of ISG15s from the indicated species with human Ub for comparison. The residue numbering is based on human ISG15. The residues in ISG15 forming the binding interface with KUPEV OTU are indicated by green bars. The seven residues focused on in this study related to differential interactions with OTUs are boxed in red. Sequence alignments were generated using CLUSTALW followed by visual inspection and adjustment [68, 69]. Initial graphics for the sequence alignments were created using the ESPript server [70].

Nairoviruses (family *Nairoviridae*), which number ~40 divided over 16 species, are globally distributed, predominantly tick-associated, arthropod-borne viruses [1, 2] (Fig 1A). Several nairoviruses cause human disease, most notably Crimean-Congo hemorrhagic fever virus (CCHFV). CCHFV is one of the most widespread hemorrhagic fever viruses [3]; infections have been reported across Africa, the Middle East, Asia, and Eastern and Southern Europe, with outbreak associated case-fatality rates ranging from 5–40%. Other nairoviruses known to cause human disease include Nairobi Sheep Disease virus (NSDV) in Africa and Asia (Asian variant Ganjam virus, GANV), Dugbe virus (DUGV) in Africa, and Erve virus (ERVEV) in Western Europe. These viruses typically cause mild fever, headache, and diarrhea [4–9].

In addition to infecting humans, many nairoviruses have been directly associated with other vertebrate hosts. CCHFV, for example, is reported to infect a wide array of mammalian species [10, 11], however disease is restricted to humans. Importantly, CCHFV maintenance and transmission relies on asymptomatic circulation among a number of hosts, including small mammals, reptiles, and livestock [10, 11]. NSDV and DUGV also infect livestock, with NSDV causing severe gastroenteritis in sheep and goats. Nairoviruses have been isolated from bats [4, 6, 12–14], and are often detected in vertebrate associated ectoparasites; Kupe virus (KUPEV), for example, was isolated from ticks infesting cattle, sheep, and goats, while others were found in ticks infesting gull nests [15–17].

Nairoviruses possess a negative sense, single-stranded RNA ((-)ssRNA) genome consisting of three segments denoted as small (S), medium (M), and large (L), that encode the viral nucleoprotein, glycoproteins, and the multifunctional L protein, respectively. Beyond the RNA-dependent RNA polymerase (RdRp), the L protein contains a viral homologue of the ovarian tumor domain protease (OTU) at the N-terminus (Fig 1B). This OTU reverses posttranslational modifications by ubiquitin (Ub) and interferon (IFN) stimulated gene product 15 (ISG15). Ub and ISG15 are conjugated to proteins (ubiquitination/ISGylation) in a process involving activating (E1), conjugating (E2), and ligating (E3) enzymes. Ubiquitination plays a key role in activation of the innate immune response, while ISGylation primarily occurs on newly synthesized proteins in response to IFN induction, making viral proteins a predominant target [18].

The OTU has been found to have an important role in immune suppression [19–22]. Reverse genetics experiments with CCHFV have demonstrated a clear connection of OTU deubiquitinase (DUB) activity with enhanced viral replication through suppression of the type I IFN response [22]. The role of OTU deISGylase activity, while not as clear, appears to be involved in promoting higher levels of L protein during later stages of CCHFV infection. ISG15 possesses a large degree of functional diversity, including a role in antiviral activity. In addition to mediating effects through ISGylation, ISG15 has been observed to function in a free form: extracellularly as a cytokine and intracellularly modulating immune responses [23–33]. Notably, the role of ISG15 appears to differ between host species, with a more pronounced antiviral effect in mice compared to humans [31].

In contrast to Ub, which is almost perfectly conserved among eukaryotes, ISG15 shows a much greater degree of diversity with sequence identities that can drop below 60% among mammals (Fig 1C). These primary structure differences may translate into tertiary structure diversity, with ISG15s from different species potentially possessing different preferred orientations of its two Ub-like domains [34–36]. Positional variation in the domains could result in different surfaces being available for protein-protein interactions, including those in regions known to interface with viral proteins [37]. Previous structural studies have identified specific elements in OTU-ISG15 interactions that might be involved in defining ISG15 substrate specificity [38]. However, a complete picture is still lacking regarding the importance of these and potentially other factors in driving ISG15 species preferences. This includes how they may differ among nairovirus OTUs.

To address this gap in understanding OTU-ISG15 interactions, 14 nairovirus OTUs spanning the virus family were assessed for their ability to cleave ISG15s from 12 different animal species. This revealed that deISGylase activity appears restricted to particular nairovirus lineages, with many nairoviruses demonstrating a lack of deISGylase activity. Within these viruses possessing deISGylase activity, a range of host ISG15 preferences are observed, with some OTUs lacking human ISG15 activity but possessing substantial activity towards other species’ ISG15s. By determining previously unresolved structures of the KUPEV and GANV OTU bound to sheep ISG15, we identified key residues required for OTU interaction with sheep ISG15. These novel structures were used to further examine key OTU and ISG15 residues responsible for OTU species preference. Comparing OTUs activity on ISG15 from multiple animal species, we identified key residues implicated in species-specific OTU-ISG15 interactions.

## Results

### Nairovirus OTUs display ISG15 species preferences

**Fig 2:**
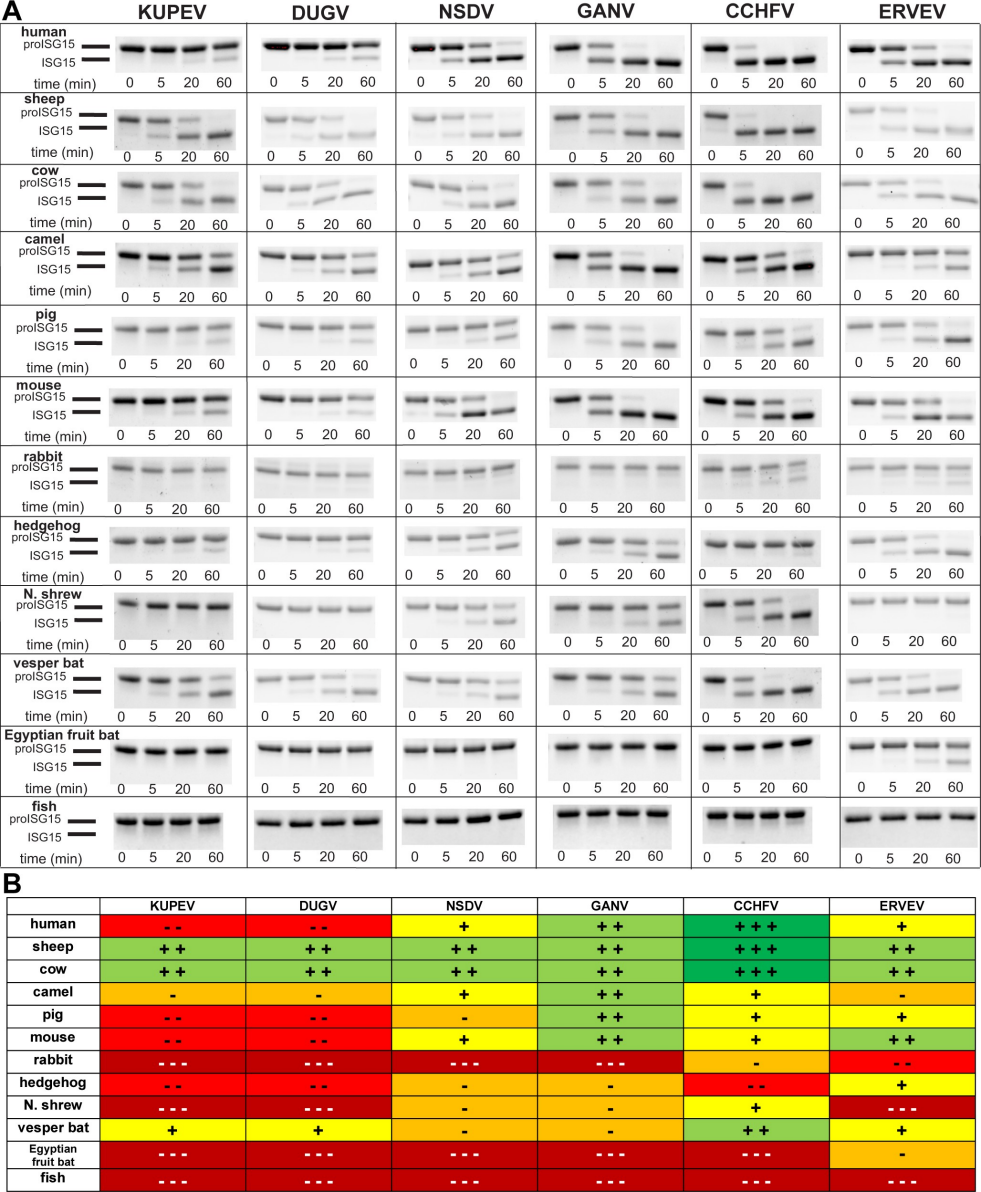
Activity of nairovirus OTUs for proISG15 from different species. (A) Cleavage of proISG15 substrates derived from different animal species by nairovirus OTUs. Each OTU was present at a 20 nM concentration and run against 10 μM of each ISG15 at 37°C. Samples were taken at the indicated timepoints and the reaction quenched in 2x Laemmli sample buffer followed by boiling at 98°C for five minutes. SDS-PAGE analysis was performed using Mini-PROTEAN^®^ TGX Stain-Free^^™^^ as described in the Materials and Methods. The substrate preference of OTUs was assessed by comparing the relative quantities of cleavage product versus unreacted substrate over the time course of the assay. (B) Summary of proISG15 cleavage assays for nairovirus OTUs presented as a heat map. Colors range from green (robust cleavage) to dark red (no cleavage).

To investigate the ability of nairovirus OTUs to cleave ISG15 from various animal species, we took advantage of the ability of viral OTUs to cleave ISG15 precursor (proISG15) into mature ISG15 [34, 38–40]. ProISG15 substrates were derived from 12 animal species and tested against a panel of 14 diverse nairovirus OTUs. Samples were taken over the course of an hour and the relative quantities of pro- versus mature ISG15 were resolved by SDS-PAGE (Fig 2 and S2A). This assay revealed that prominent deISGylase activity is largely restricted to nairoviruses in the lineage that includes CCHFV such as Thiafora virus, NSDV, and DUGV [41, 42], while no ISG15 cleavage is observed using OTUs from many of the viruses outside of these lineages. However, phylogenetic relatedness, or OTU similarity, of viruses does not always equate to comparable deISGylase activity (Fig 1A). While the closely related KUPEV and DUGV possess almost identical cleavage profiles, the same cannot be said for NSDV and GANV. Despite being a variant of the same virus, GANV possesses enhanced activity towards human, camel, pig, and mouse proISG15 (Fig 2B).

Interestingly, some OTUs that possess weak or undetectable activity for human proISG15 possess substantial activity for ISG15 of other species. For instance, the OTUs from KUPEV and DUGV have limited activity for human proISG15, but efficiently cleave sheep and cow proISG15s and show moderate activity towards camel and vesper bat proISG15s. Likewise, these OTUs poorly cleave human ISGylated host substrates in a cell-based assay (S2B Fig). Even within the same order of mammals, differences between ISG15s can lead to substantial differences in their ability to be engaged by OTUs. For instance, vesper bat and Egyptian fruit bat ISG15s share only 64% sequence identity. Accordingly, they have completely different interaction profiles. While the proISG15 from vesper bat is processed by several of the OTUs, the Egyptian fruit bat proISG15 is only cleaved by ERVEV. With the exception of fish proISG15, which shows no sign of cleavage by any of the OTUs, most of the other proISG15s show varying susceptibility to processing by the nairovirus OTU panel. Altogether, these data suggest that even small sequence differences may play a role in tuning OTU activity, resulting in a wide range of OTU-ISG15 interaction profiles.

### Structures of KUPEV OTU and GANV OTU bound to sheep ISG15 provide insight into the role of the OTU selectivity pocket on deISGylase activity

The proISG15 cleavage assay raised the question of why certain ISG15s (e.g., sheep and cow) are cleaved efficiently by various OTUs, while others (e.g., human and N. shrew) are not (Fig 2B). As a first step to answer this question, we aimed to gain structural insights into OTU interactions with sheep ISG15 by generating never before elucidated crystal structures of sheep ISG15 in complex with KUPEV and GANV OTU, and by identifying key OTU and ISG15 binding residues. Sheep ISG15 was derivatized into a suicide inhibitor with propargylamine and incubated with KUPEV and GANV OTUs to form covalent complexes that could be used for X-ray crystallography. This yielded an atomic resolution structure of KUPEV OTU bound to the C-terminal domain of sheep ISG15 (C-sheep ISG15) solved to 2.06 Å and a low-resolution structure of GANV OTU bound to full length sheep ISG15 to 3.15 Å (Table 1).

**Table 1:** Data collection and refinement statistics.

	KUPEV OTU-C-sheep ISG15 (PDB entry 6OAR)	GANV OTU-sheep ISG15 (PDB entry 6OAT)
**Data collection**		
Space group	P2_1_2_1_2_1_	P6_1_
Wavelength (Å)	1	1
Cell dimensions		
*a*, *b*, *c* (Å)	41.8, 158.8, 171.0	55.0, 55.0, 494.8
α, β, γ (°)	90, 90, 90	90, 90, 120
Resolution (Å)	50.00–2.06 (2.11–2.06)^†^	50.00–3.15 (3.20-3.15)^†^
*R*_pim_	0.049 (0.450)	0.074 (0.523)
*CC*_*1/2*_	0.994 (0.699)	0.994 (0.662)
*I* / σ*I*	15.9 (1.51)	11.2 (1.1)
Completeness (%)	99.8 (99.9)	85.1 (82.0)
Redundancy	5.1 (4.6)	6.8 (7.0)
**Refinement**		
Resolution (Å)	41.29–2.06 (2.14–2.06)	39.51–3.15 (3.20-3.15)
No. reflections	70,367	12,240
*R*_work_ (%)/ *R*_free_ (%)	17.7/21.7	25.3/30.5
No. atoms		
Protein	7532	4912
Ligand/ion^‡^	16	8
Water	359	0
*B*-factors		
Protein	45.79	117.57
Ligand/ion^‡^	38.02	79.33
Water	45.26	---
R.m.s. deviations		
Bond lengths (Å)	0.005	0.003
Bond angles (°)	0.55	0.61

^†^Values in parentheses denote the highest resolution shell

^‡^Includes the propargylamine linker

**Fig 3:**
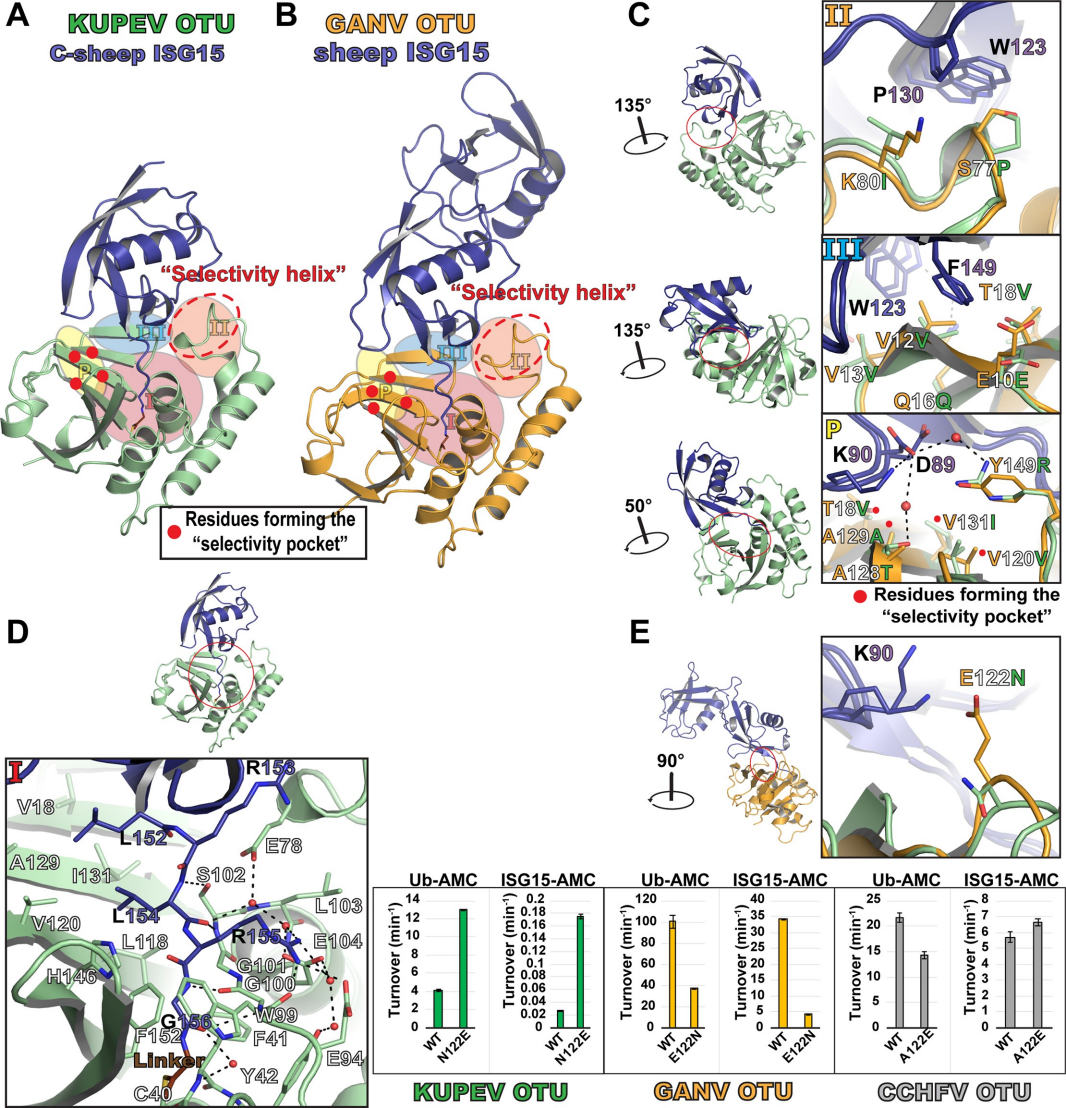
Structures of the KUPEV and GANV OTUs in complex with sheep ISG15. (A) A cartoon rendering of the KUPEV OTU (green) bound to the C-terminal domain of sheep ISG15 (purple). The propargylamine molecule covalently linking the ISG15 and OTU is shown in brown. The regions of the OTU forming the interface with sheep ISG15 are indicated by a shaded background. The approximate locations of residues forming the selectivity pocket are indicated by red dots. The secondary structure for KUPEV OTU as calculated by the DSSP server is indicated [71]. (B) A cartoon rendering of the GANV OTU (orange) bound in complex with sheep ISG15 (purple), annotated as in (A). (C-D) Closeup views of major interactions within the three main regions (I-III) and peripheral regions (P) of the KUPEV/GANV interface with sheep ISG15. Black dashes indicate interatomic distances ≤3.5 Å between atoms capable of forming electrostatic pairs. (E) A closeup of residue 122 in the KUPEV and GANV OTUs, with the impact of mutating this residue measured by activity towards Ub-AMC and human ISG15-AMC. Values are the mean ± standard deviation of two independent experiments.

Examination of the high resolution KUPEV and low resolution GANV OTU-sheep ISG15 structures reveals them to possess the familiar overall mode of OTU-substrate binding, in which the OTU binds to the C-terminal domain of ISG15 by a mix of hydrophobic and electrostatic interactions (Fig 3A and 3B, [38, 43–46]). The main areas of interaction can be divided into three major regions of the OTU surface (Fig 3 and S3A, [43, 45, 46]). Region I surrounds the OTU active site and interacts with the tail of sheep ISG15 through electrostatic interactions (Fig 3D, Panel I). Regions II and III of the OTU surface encompass the α3 “selectivity helix” and beta sheets 1, 3, and 4, respectively, and work in tandem to create the major hydrophobic interface with sheep ISG15 outside of the ISG15 tail. On the sheep ISG15 side, the interface with the OTUs is formed by W123, P130, and F149 (Fig 3A–3C Panels II and III). In addition to these central hydrophobic interactions, there are also some contributing peripheral electrostatic interactions, including both direct and water-mediated components (S3B Fig).

One of the most noteworthy interactions is that of sheep ISG15 D89 forming water-mediated interactions with both KUPEV OTU T128 and R149 (Fig 3C, Panel P). Interestingly, this results in D89 pointing away from the interface, contrasting with previous observations for this residue position in OTU-Ub structures and CCHFV OTU-human ISG15 structures. In the case of OTU-Ub structures, the analogous residue to sheep ISG15-D89 is L8 which inserts into a OTU “selectivity pocket” whose hydrophobic nature is critical for efficient Ub binding [41]. Considering the selectivity pocket in KUPEV is lined by hydrophobic residues V18, V120, A129, and I131, the adoption of a more solvent-exposed confirmation of the charged aspartate in sheep ISG15 is understandable. GANV and KUPEV OTUs interact with sheep ISG15 in a highly similar manner, including how GANV OTU accommodates sheep ISG15 D89 outside of the selectivity pocket (Fig 3C panel P). Given that both OTUs of GANV and KUPEV exhibit robust deISGylation activity towards sheep ISG15, this highlights that accommodation of D89 in the selectivity pocket is not necessary for robust OTU deISGylase activity. Hence, while the hydrophobicity of the selectivity pocket is critical and generally predictive for Ub binding to nairovirus OTUs, an analogous predictive role for the pocket in ISG15 binding is not likely to be as pervasive.

### OTU residue 122 influences overall OTU activity

**Fig 4:**
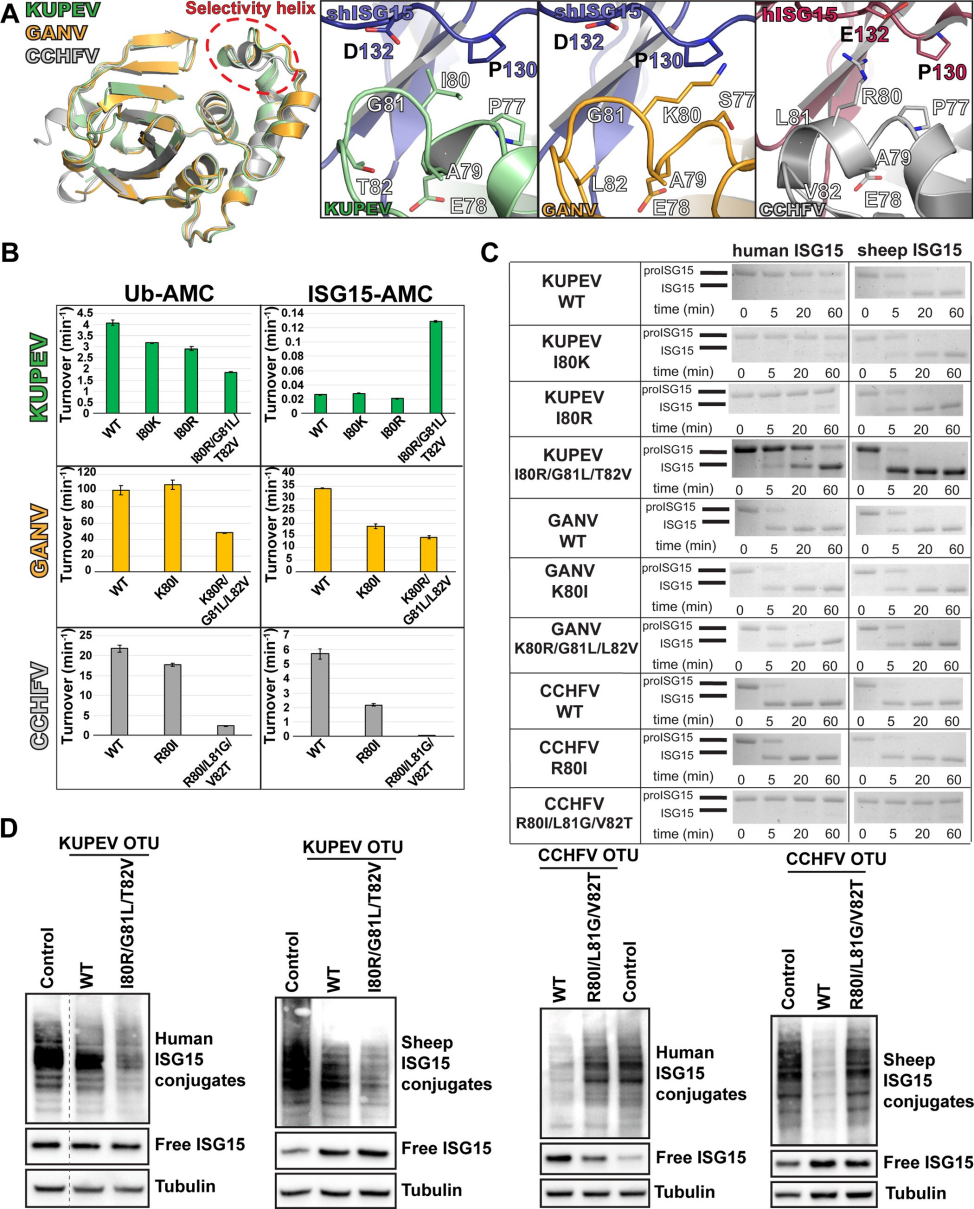
Targeting the OTU “selectivity helix” to alter ISG15 preference. (A) Overlay of the KUPEV, GANV, and CCHFV OTUs. The OTU “selectivity helix” is indicated by a red oval, with closeup views of the helix in each structure shown to the right. (B) Impact of single or multiple mutations within the helix on the activity of the KUPEV, GANV, and CCHFV OTUs towards Ub-AMC and human ISG15-AMC. Values represent the mean ± standard deviation of two independent experiments. (C) Cleavage assays of OTU mutants with human and sheep proISG15. Samples from each timepoint were run on BioRad Mini-PROTEAN® TGX^™^ gels and visualized by Coomassie staining. (D) Western blot analysis of OTU activity on conjugated human or sheep ISG15. Cell lysates containing conjugated human or sheep ISG15 was incubated with purified OTUs, and deISGylase activity was assessed by Western blot.

The most noticeable difference in the interface of the OTUs from KUPEV and GANV with sheep ISG15 is located at position 122. For GANV OTU, this position is occupied by a glutamate that is in proximity to interact with sheep ISG15’s K90. In other ISG15s this position is universally a lysine/arginine, and in Ub a threonine. In the KUPEV OTU residue 122 is an asparagine residue, while CCHFV is unique among OTUs with an alanine at this position (Fig 1A). These residues would not have the same potential to interact with ISG15 or Ub substrates as E122 in GANV OTU, suggesting this may partially account for the observed enhanced activity of GANV OTU towards Ub and human ISG15-7-amido-4-methylcoumarin (AMC) substrates [41]. To confirm this, E122 in GANV OTU was mutated to an asparagine, and the corresponding positions in the KUPEV and CCHFV OTUs to glutamate. As anticipated, the GANV OTU E122N mutant reduced activity for human ISG15-AMC almost 10-fold, while KUPEV OTU N122E boosted activity more than 6-fold relative to WT (Fig 4B). The CCHFV OTU A122E mutant had a modest effect with just a marginal increase in activity, suggesting that in some OTUs other features may dictate the relative importance of this residue.

### The OTU α3 selectivity helix contributes to OTU specificity for human and sheep ISG15

**Fig 5:**
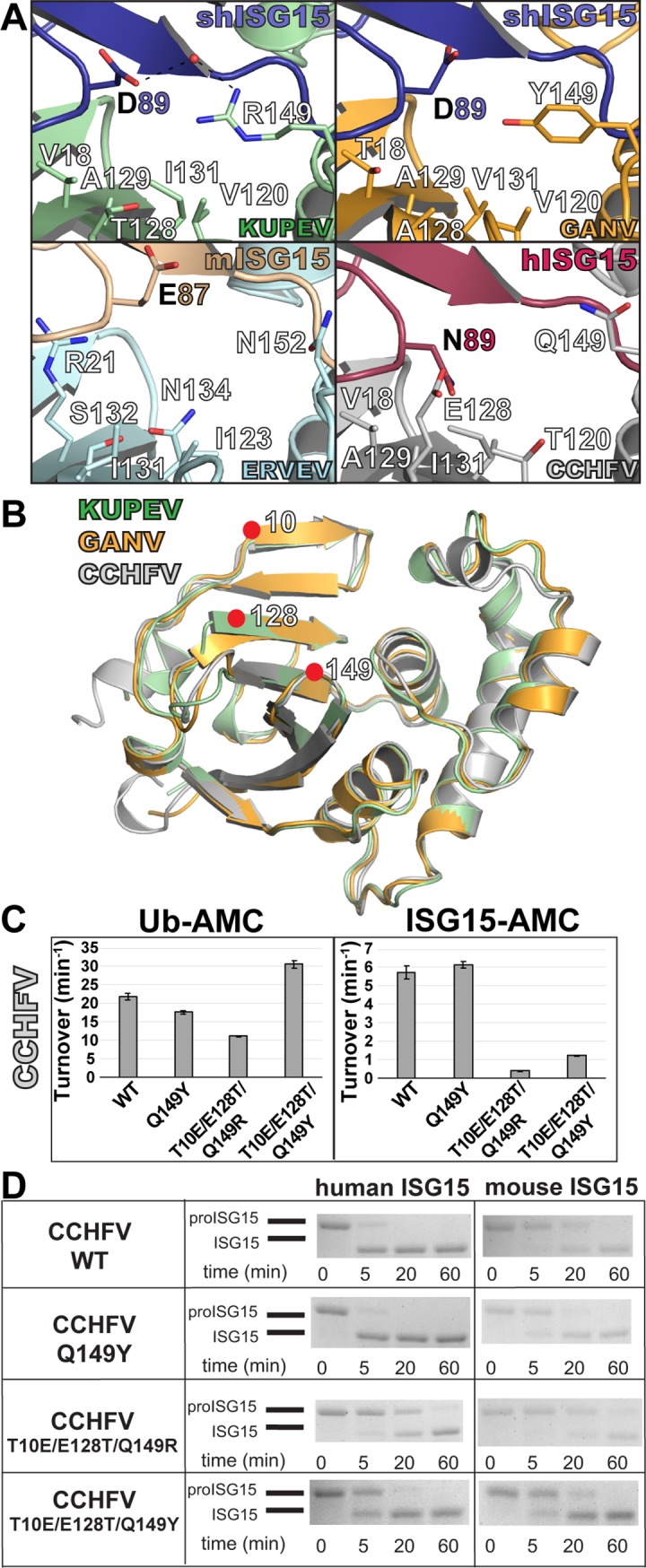
Selectively altering CCHFV OTU interactions with human ISG15 and mouse ISG15. (A) Closeup views of the structural environment encompassing residues 128 and 149 (131/152 in ERVEV) of the OTUs is shown. (B) Overlay of the KUPEV, GANV, and CCHFV OTUs with the targeted sites shown by red dots. (C) Impact of mutations on the activity of CCHFV OTU towards Ub-AMC and human ISG15-AMC. Values shown for the AMC assays represent the mean ± standard deviation of two independent experiments. (D) Impact of mutations on the activity of CCHFV OTU towards human proISG15 and mouse proISG15. Samples from each proISG15 assay timepoint were run on BioRad Mini-PROTEAN® TGX^™^ gels and visualized by Coomassie staining.

The ability to increase GANV and KUPEV OTU activities towards human ISG15 with a single amino acid change led us to probe whether other OTU structural motifs could play a role in altering OTU activities towards other ISG15 species. We therefore assessed the potential structural features delineating OTU preference for human versus sheep ISG15, using the KUPEV, GANV and CCHFV OTUs. KUPEV OTU possesses low activity for human proISG15, but a high activity for sheep proISG15, whereas the GANV and CCHFV OTUs possess similar activities for either ISG15 (Fig 2). Examination of the OTU surface interacting with human and sheep ISG15 suggested that the OTU α3 selectivity helix [44] could play a role in differential specificity (Fig 5). This helix, particularly residue 80, is positioned where interactions with residue 132 in ISG15, a glutamate in human versus aspartate in sheep, may be able to influence binding. In GANV and CCHFV residue 80 is a lysine and arginine, respectively, which are both flexible and capable of forming an electrostatic interaction. In KUPEV OTU this residue is an isoleucine, creating the potential for a steric clash with the longer glutamate in human ISG15 compared to aspartate in sheep ISG15 (Fig 4A).

To examine whether the selectivity helix might be responsible for differentiating human and sheep ISG15, mutations were introduced to each OTU at residue 80. Additionally, due to the difference in the position of the helix as a result of a glycine at position 81 in KUPEV and GANV versus a leucine in CCHFV, additional mutants were made to examine the impact of this helix’s localization by converting residues 80–82 in KUPEV and GANV to the corresponding ones in CCHFV, and vice versa. To assess the impact of these changes, each mutant was tested for activity towards human ISG15-AMC, human proISG15, and sheep proISG15, as well as Ub-AMC to assess the effect on activity beyond ISG15 (Fig 5). In KUPEV OTU, residue 80 alone appears not to have a significant impact on ISG15 cleavage. In contrast, the KUPEV helix mutant (I80R/G81L/T82V) demonstrates a 5-fold increase in human ISG15-AMC activity while reducing Ub-AMC activity by half. This trend is also seen on sheep and cellular proteins ISGylated proteins with sheep or human ISG15, demonstrating that these changes seen with pure enzymatic substrates do carry over to cellular substrates (Fig 5). This increase in ISG15 activity is reflected in both human and sheep proISG15 cleavage, suggesting that this helix is important for human ISG15 activity and ISG15 activity in general.

For GANV OTU, the K80I mutant reduces human ISG15-AMC activity by half but does not affect Ub-AMC, while the helix mutant reduces both by approximately half. Interestingly, this does not seem to be completely reflected between species in the proISG15 assay. Specifically, only the GANV helix mutant’s ability to cleave human proISG15 appears to be affected, not the sheep counterpart. However, this differs for CCHFV OTU. Alteration of the CCHFV helix mutant within this region has a drastic impact on its ability to cleave Ub and ISG15 substrates. The helix mutant obliterated human ISG15-AMC activity while reducing the activity towards Ub-AMC almost 10-fold, and almost no cleavage of either human or sheep proISG15 and host ISGylated proteins were observed (Fig 5). The R80I mutant, on the other hand, was more selective, reducing human ISG15-AMC activity by more than half while only lowering Ub-AMC activity less than 20%. In addition, this mutant showed a differential impact in the proISG15 assay, with a noticeable reduction in human proISG15 cleavage while sheep proISG15 was not detectably impacted. This indicates that the relative activity towards human and sheep ISG15 can be influenced by the selectivity helix.

### Shifting OTU specificity between human and mouse ISG15

Beyond altering OTU specificity for ISG15s from human and sheep, the possibility to enhance OTU specificity for mouse ISG15 was investigated. To assess which OTU residues are important for mouse ISG15 preference, we compared OTUs in complex with mouse, sheep and human ISG15. This suggested that OTU accommodation of mouse ISG15 residue 87 (residue 89 in sheep and human ISG15) may play an important role (Fig 5A). This residue is pointed away from the OTU-ISG15 interface in KUPEV and GANV OTUs bound to sheep ISG15, and ERVEV OTU bound to mouse ISG15. Both KUPEV and GANV OTUs are able to interact with D89 in sheep ISG15 through water-mediated electrostatic interactions with an arginine (R149) or tyrosine (Y149) residue. This results in the possibility that the longer E87 in mouse ISG15 could form a direct electrostatic interaction with these residues if they were present in CCHFV OTU. However, CCHFV possesses a glutamine (Q149) that would be less suitable to form this interaction with mouse ISG15. To assess whether the formation of a mouse ISG15 E87 –OTU Y149 interaction would be beneficial to enhancing an OTU activity towards mouse ISG15, a Q149Y mutant of CCHFV OTU was generated (Fig 5). As expected, this mutant did not affect OTU activity on human ISG15-AMC, and OTU activity towards mouse proISG15 modestly but noticeably improved, with increased product formation at 5 minutes (Fig 5C and 5D).

In order to generate an OTU that specifically preferred mouse ISG15 over human ISG15, additional residues were sought that simultaneously decreased CCHFV OTU activity toward human ISG15. CCHFV residue E128 stood out as a candidate, since E128 is expected to facilitate the interaction with human ISG15 N89 but likely not with mouse ISG15 E87 (Fig 5A). Therefore, this residue was mutated to threonine (E128T) and combined with either a Q149R or Q149Y mutation to promote mouse ISG15 activity. Due to the documented impact of the E128T in reducing Ub activity as well, an additional mutation was added (T10E) that is favorable for Ub in an attempt to compensate [44]. Analysis of the T10E/E128T/Q149R mutant revealed that OTU activity towards human ISG15-AMC activity is reduced by more than 90%, while Ub-AMC activity remained ~50% of the WT OTU. However, activity of this triple mutant towards mouse proISG15 suffered a small reduction, albeit not to the same degree as for human proISG15. In contrast, the T10E/E128T/Q149Y triple mutant reduced activity for human ISG15-AMC by almost 80%, while boosting Ub-AMC activity by ~40%. This mutant also had markedly reduced cleavage observed with human proISG15, with some substrate still remaining after 20 minutes. Cleavage of mouse proISG15 is boosted relative to the wildtype OTU and closely resembles what is achieved by the Q149Y mutation alone. This puts the activity of this triple mutant towards mouse proISG15 to levels just slightly weaker than the activity shown with human proISG15. As a result, this further highlights the feasibility of significantly altering an OTU’s activity toward an ISG15 of one species at the expense of another with just a few mutations.

### Identification of ISG15 residues responsible for OTU species specificity

**Fig 6:**
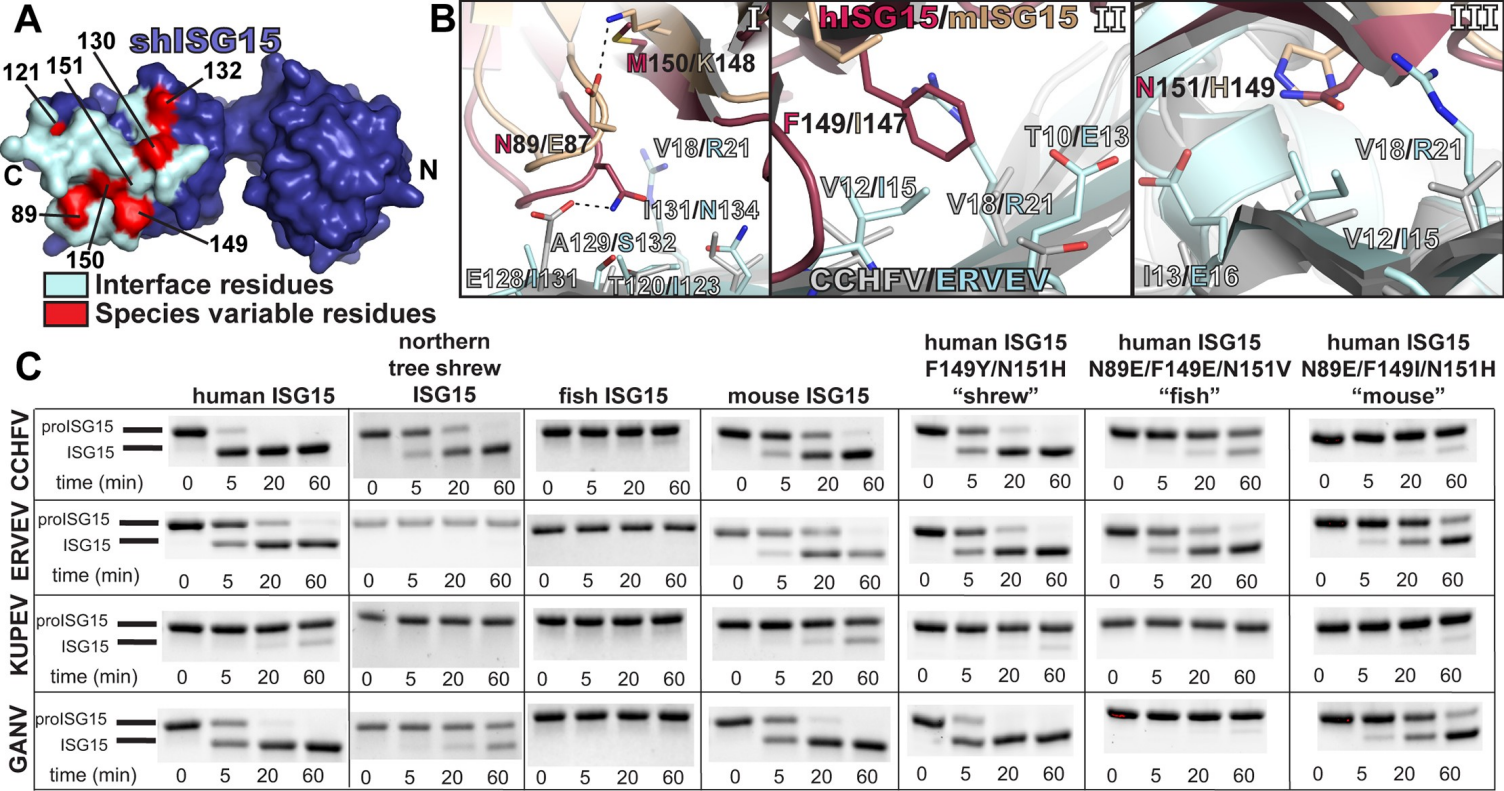
Molecular contributors to species-variable ISG15 interactions with nairovirus OTUs. (A) Surface rendering of sheep ISG15 (purple) with the residues forming the interface shown in light teal. The locations of residues 89, 149, and 151 that have been previously suggested as responsible for species-specific interactions are indicated. (B) Molecular environment of the CCHFV OTU-human ISG15 (PDB ID 3PHX) and ERVEV OTU-mouse ISG15 (PDB ID 5JZE) structures surrounding residues 89 and 149–151 (87 and 147–149 in mouse ISG15). (C) Cleavage assays of the CCHFV, ERVEV, KUPEV, and GANV OTUs with mutant human proISG15 constructs. Mutations were introduced to human proISG15 at residues 89, 149, and 151 to match those in northern tree shrew, fish, or mouse ISG15. Samples from each timepoint were run on BioRad Mini-PROTEAN^®^ TGX Stain-Free^^™^^ gels and visualized as described in the Materials and Methods.

The structures of sheep ISG15 in complex with the OTUs from KUPEV and GANV further reinforce that nairovirus OTU interactions with ISG15 are confined to a well-defined region of the ISG15 surface, formed by the C-terminal ISG15 residues (Fig 1C, Fig 3, Fig 6A) [37, 38, 43, 46]. Several of the ISG15 residues interacting with OTUs are highly conserved, restricting the probable contributors of species preference to just a handful of variable residues (Fig 1C). Previous OTU-ISG15 structures identified ISG15 residues 89 and 149–151 (87 and 147–149 in mouse ISG15) as key residues involved in OTU-interactions. Since these residues are not conserved, they have the potential to affect OTU species preference (Fig 6A and 6B) [38].

To test the influence of these four positions on ISG15 species preference, mutations were introduced to the proISG15 constructs and assessed for their impact on cleavage by the OTUs (Fig 6C). First, we mutated human proISG15 to resemble ISG15 from species that CCHFV OTU does not efficiently cleave. Human ISG15 was mutated at these positions to resemble shrew (F149Y/N151H), fish (N89E/F149E/N151V), or mouse (N89E/F149I/M150K/N151H). All of these ISG15 mutants are less efficiently cleaved by CCHFV OTU, especially the fish-like and mouse-like mutants. In addition, the fish-like ISG15 mutant demonstrated a complete lack of cleavage when it was tested with KUPEV and GANV OTUs.

**Fig 7:**
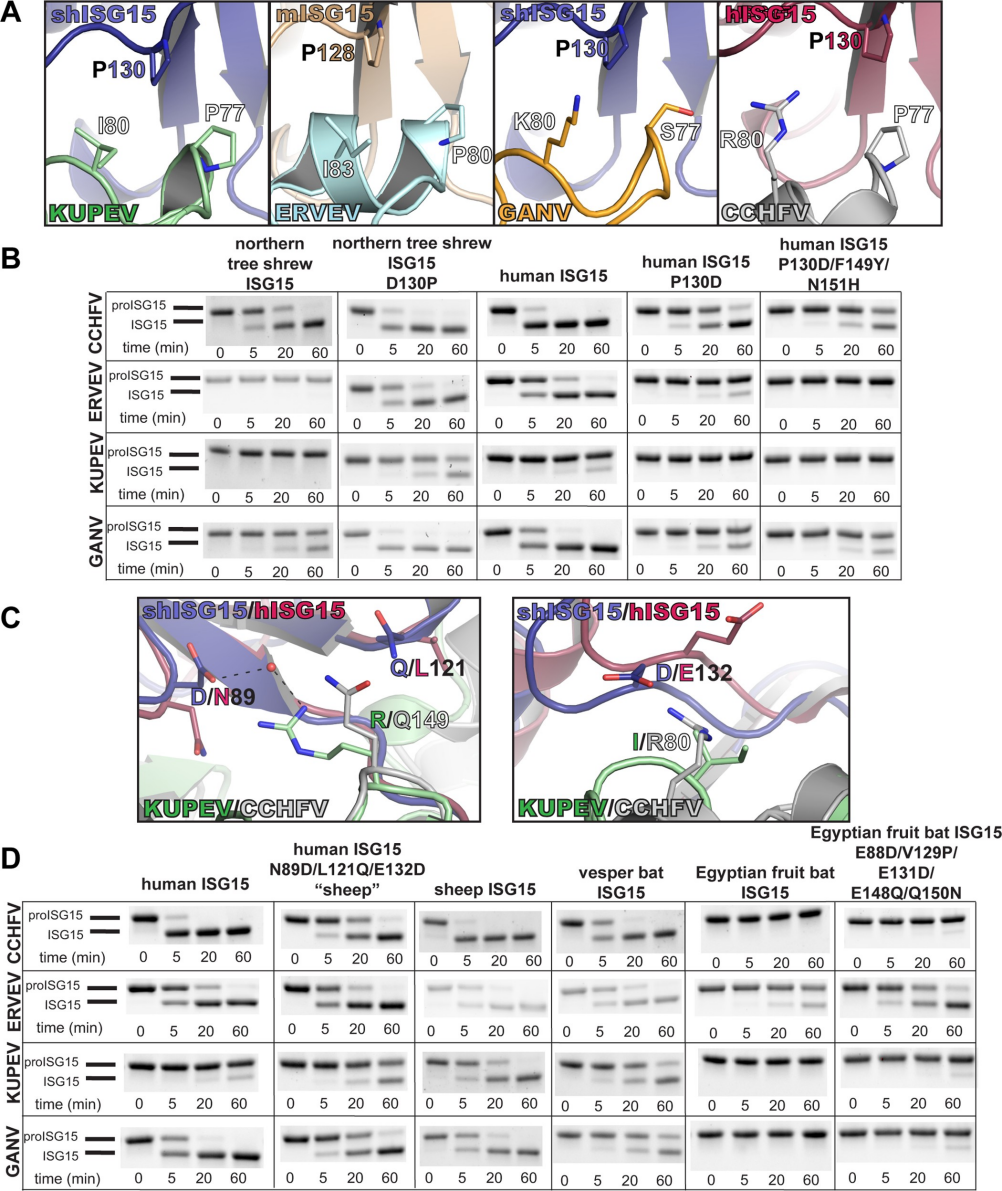
The impact of ISG15 residues 130, 121, and 132 on species-specific OTU-ISG15 interactions. (A) Comparison of KUPEV OTU-sheep ISG15, ERVEV OTU-C-mouse ISG15 (PDB ID 5JZE), GANV OTU-sheep ISG15, and CCHFV OTU-human ISG15 (PDB ID 3PHX) interactions between residue 130/128 of ISG15 and residues 77–80 of the OTU highlighting the variable sensitivity of this region. Cleavage assays show the relative impact of mutations in human proISG15 and northern tree shrew proISG15 on reactions with the CCHFV, ERVEV, KUPEV, and GANV OTUs. (B) KUPEV OTU-C-sheep ISG15 and CCHFV OTU-C-human ISG15 interactions at points that differ between sheep ISG15 and human ISG15. Cleavage assays show the impact of performing residue swaps in human proISG15 and Egyptian fruit bat proISG15 on reactions with CCHFV, ERVEV, KUPEV, and GANV. Samples from each timepoint were run on BioRad Mini-PROTEAN^®^ TGX Stain-Free^^™^^ gels and visualized as described in the Materials and Methods.

In contrast, mutating these four variable ISG15 positions did not exert the expected effect for every OTU. In particular, the shrew-like and fish-like ISG15 mutations did not impact ERVEV OTU activity. Similarly, KUPEV and GANV OTU activity on the shrew-like ISG15 mutant seemed to be unaffected. This suggests that other factors may be at play that have a more important role in some OTU-ISG15 interactions. Re-examination of the ERVEV OTU-mouse ISG15 structure in conjunction with the KUPEV OTU-sheep ISG15 structure revealed aspects of the ERVEV and KUPEV OTUs that may account for these observations. Specifically, the OTU hydrophobic cleft formed by KUPEV P77/I80 and ERVEV P80/I83 may be critical in binding ISG15 P128/130 (Fig 7A). Although proline is the most common residue at this position in ISG15s, in the case of northern tree shrew this residue is an aspartate (D130). This aspartate pointing into the hydrophobic surface created by KUPEV I80 and ERVEV I83 would likely interfere with binding of northern tree shrew ISG15 D130. Contrasting the hydrophobic residues encoded by KUPEV OTU (I80) and ERVEV OTU (I83), the GANV and CCHFV OTUs contain residues (K80 and R80 respectively) that result in a less stringent ISG15 binding environment, and are therefore better able to accommodate the northern tree shrew ISG15 D130. To test the importance of ISG15 residue 130, we mutated northern tree shrew proISG15 (D130P). Mutating this site alone is able to introduce or enhanced cleavage of shrew proISG15 by all the OTUs tested (Fig 7B). Conversely, the opposite mutation in human proISG15 (P130D) was able to reduce or eliminate cleavage by all tested OTUs. Combining the P130D with other mutations, F149Y and N151H in human proISG15 to generate a “shrew-like” ISG15 further reduced OTU activity, demonstrating that these sites act synergistically to confer ISG15 species specificity among OTUs.

The previous four highlighted residues (89, 149–151) were identified principally due to their potential for steric clashes, or secondary structural changes, within the OTU–ISG15 interface [38]. However, the influence of position 130 appears to be more modulated by affinity on the edge of the OTU–ISG15 interface. This raised the question of whether there may be other less obvious residues on the periphery of the OTU-ISG15 interface that can influence OTU activity towards ISG15s. This could potentially explain how OTUs appear to differentiate between human and sheep ISG15. These two ISG15s are highly similar at the five positions identified so far to be drivers of OTU species specificity towards ISG15s. Comparison of the newly available structures of KUPEV with sheep ISG15 and CCHFV with human ISG15 suggested two other residues that could potentially contribute to the OTU-ISG15 interface: L121 and E132 in human ISG15 versus Q121 and D132 in sheep ISG15 (Fig 7C). The location of ISG15 residue 121 suggests a transient interaction could occur with an OTU, such as from KUPEV, that possesses an arginine at position 149. At position 132 in ISG15, most known ISG15s possess a glutamate at this position. Only a few ISG15s, like from sheep, have an aspartate at that position. This difference could potentially add a steric, or electrostatic component that could interfere with compatibility of an OTU’s selectivity helix, particularly at OTU position 80. To investigate the relative contribution of these ISG15 residues to the ISG15-OTU species preference, we introduced three sheep-like mutations in human ISG15 (N89D/L121Q/E132D). These mutations were expected to increase human ISG15’s susceptibility for KUPEV and ERVEV OTU. These mutations resulted in a modestly increased human proISG15 cleavage by KUPEV OTU, while GANV and CCHFV cleaved the sheep-like human ISG15 mutant less efficiently (Fig 7D). Taken together, this shows that five ISG15 residue positions (89, 130, and 149–151) strongly contribute to species-specific OTU interactions, while at least two further residue positions (121 and 132) play additional roles.

To further assess the crucial role of these ISG15 residues in determining OTU species preference, these sites in vesper bat versus Egyptian fruit bat ISG15 were compared. These two ISG15s provide a stark contrast in the activity that OTUs generally exhibit towards them, with moderate to high cleavage observed for vesper bat, while only ERVEV is able to cleave Egyptian fruit bat proISG15 (Fig 2). As would be expected, these two ISG15s differ at five out of the seven identified residue positions (Fig 1B). To test the influence of these residues in distinguishing the two bat species, these sites were mutated in Egyptian fruit bat proISG15 to match the corresponding residues in vesper bat proISG15. Though modestly, these mutations were able to introduce cleavage of Egyptian fruit bat proISG15 by KUPEV, GANV, and CCHFV, and enhanced the cleavage by ERVEV (Fig 7D). Overall, these assays demonstrate that shifting ISG15 substrate preferences can be accomplished through specifically mutating a particular set of residues, and that this can have a key role in species-specific cleavage activity patterns.

## Discussion

Here we investigated nairovirus ISG15 species preference by assessing deISGylase activity for 14 nairovirus OTUs against ISG15s from 12 different animal species. Surprisingly, we found that only a portion of nairoviruses appear to display robust deISGylase activity, and these OTUs display a clear preference for ISG15 from distinct species. Using new structures of KUPEV and GANV OTUs bound to sheep ISG15, and comparing them to previously reported structures of CCHFV and ERVEV OTUs bound to human and mouse ISG15, respectively [38, 43, 46], we identified residues in the OTU-ISG15 interface critical for this species-specificity. Furthermore, using structure-guided mutational analyses, we identified seven ISG15 residues primarily responsible for the observed species-specificity of nairovirus OTUs.

Our study raises questions on the ability of OTUs to adapt to a host species, and what drives the specificity of OTUs for particular species’ ISG15. Most nairoviruses are known to persistently infect ticks, which serve as vector and reservoir, while vertebrates are amplifying hosts developing transient infection. Therefore, the evolution of nairoviruses is likely influenced by both arthropod and vertebrate hosts. Since ticks do not encode ISG15, OTU deISGylase activity was likely acquired as a result of nairovirus interactions with mammalian hosts and may therefore be important for host tropism and maintenance in nature. This is supported by our finding that deISGylase activity is only present in nairoviruses associated with mammalian host species, including sheep and cattle. Notably, we found that many OTUs display robust activity towards ISG15s from these species (Fig 2). Efficient ISG15 activity could increase and/or prolong the viremic period in mammals, therefore enhancing transmission to other ticks, forming an overall selective advantage. Nairovirus species devoid of deISGylase activity may preferentially infect vertebrates lacking ISG15, such as birds, or are primarily restricted to arthropod hosts. Although ticks lack ISG15, they possess other Ub-like proteins, such as SUMO and Nedd8 [47], which could drive OTU evolution in adaptation to some arthropods. Of note, not all nairovirus species possess an OTU; this feature is restricted to viruses of the tick-associated *Orthonairovirus* genus, while nairoviruses associated with millipedes and spiders lack this domain. This supports the notion that the OTU was acquired during nairovirus evolution to counter tick or vertebrate antiviral responses.

Currently, how variation in nairovirus OTU activity on ISG15 relates to disease susceptibility in a particular host is unclear. Despite showing similar preferences for human, sheep, and cow ISG15, CCHFV causes severe disease in humans but is asymptomatic in livestock. Similarly, NSDV/GANV can cause deadly illness in sheep and goat populations, while cattle and other livestock are refractory to infection [14]. A recent study demonstrated that species-specific sequence differences in ISG15 can impact viral tropism. The influenza B nonstructural protein 1 is able to sequester and counter the antiviral effects of human ISG15, but not mouse ISG15. These species-specific differences in ISG15 have been suggested to contribute to influenza B’s limited host tropism [35, 48–51]. Along similar lines, coronavirus deISGylases also show biochemical sensitivity to ISG15 species-species differences, which has been suggested to potentially contribute to the preferred host ranges of these viruses [34, 36, 39]. Of course, ISG15 represents only one aspect of the virus-host interface, and other factors contribute to disease. In addition, the importance of ISG15 in the immune response varies between different species. For example, mouse ISG15 plays a central role, whereas human ISG15 is less crucial for antiviral immunity.

ISG15 from different species have been observed to ISGylate substrates with varying degrees of efficiency, and the key residues driving these differences are also involved in the OTU binding interface [37, 52]. Similarly, the primary cellular deISGylase, USP18, interacts with the same general surface of ISG15 as OTUs [37, 53, 54]. Interestingly, the regions of USP18 that interface with ISG15 are highly conserved (S4 Fig), suggesting the potential for ISG15 variability to create species-specific dynamics in these interactions. This has been suggested to be a contributing factor to differences in the antiviral effect of ISG15 when comparing mice and humans [31]. In addition to its deISGylase activity, USP18 also negatively regulates IFN signaling through association with the IFN receptor. Stronger association of human USP18 and ISG15 enhances the stability of USP18, prolonging this inhibitory effect in humans, but not in mice. Thus, considering the varying contributions of ISG15 to antiviral immunity, it cannot be assumed that equivalent OTU deISGylase activities always translate to equivalent presentations of illness between species.

The observed trends in OTU-ISG15 preferences provide a strong foundation for assessing virus-host interactions, and could serve as a marker for viral host range. Virus-host interactions may be predicted from ISG15 sequence alone, possibly allowing for the identification of previously unknown hosts involved in the enzootic maintenance of nairoviruses. In addition, these new structures and biochemical data may provide insights into the direction in which a given nairovirus is adapting. Our data demonstrates that only a few changes are necessary for a virus to adapt to ISG15 of a different species, potentially affecting host range or disease manifestation/pathogenesis. Testing mutant variants of nairovirus OTUs may help anticipate the degree to which it would have to adapt to human hosts and provide insights into the potential threat posed by the virus.

In addition to determining virus-host interactions, identification of key residues impacting OTU-ISG15 interactions may allow us to capitalize on the role of ISG15 in conferring susceptibility to disease. This could contribute to the development of novel animal models of disease. Animal models to study nairovirus disease, specifically CCHFV, have been largely limited to use of immunocompromised animals such as Stat-1^-/-^ or IFNAR^-/-^ mice [55, 56]. While a non-human primate model has been recently described for CCHFV [57], immunocompetent small animal models to study disease and potential treatments are lacking. Modification of ISG15 could be a more conservative and targeted approach to alter susceptibility to particular nairoviruses in animals for disease research. Alternatively, this approach could be employed to modify ISG15 in agriculturally important animals to make them more resistant to disease.

The role of OTU-ISG15 interactions during nairovirus infection have remained largely in obscurity. This is in part due to the mystery that has enshrouded ISG15 function, including the potential that it differs between species, and the lack of clarity on the full impact of the OTU in nairovirus infections. Here we expand the knowledge of OTU interactions by showing that viral OTU deISGylase activity levels are associated with particular nairovirus lineages, and that there is a direct relationship between preferred species’ ISG15 and reported host tropism. Furthermore, by investigating OTU-ISG15 interactions using novel structures, we identify specific residues in both the OTU and ISG15 that primarily drive these preferences. These findings can be used to guide future studies on the function of ISG15 in countering viral infections, and in turn, it’s role in viral ecology and disease.

## Materials and methods

### Constructs, expression and purification of OTUs and ISG15s

The OTUs of CCHFV, DUGV, ERVEV, NSDV, GANV, TAGV, QYBV, FARV, HpTV-1, ISKV, LPHV, DGKV, HAZV, and KUPEV were constructed, expressed, and purified as previously described [38, 41, 44, 45]. For ISG15s, those in the pro form from human (*Homo sapiens*; Accession: AAH09507.1), mouse (*Mus musculus*; Accession: AAB02697.1), sheep (*Ovis aries;* Accession: AF152103.1), dromedary camel (*Camelus dromedarius*; Accession: XP_010997700.1), northern tree shrew (*Tupaia belangeri*; Accession: AFH66859.1), vesper bat (*Myotis davidii*; Accession: ELK23605.1), and fish (*Oplegnathus fasciatus*; Accession: BAJ16365.1) were constructed, expressed and purified as previously described [38].

Similarly to the previously reported proISG15s, the constructs of proISG15s originating from pig (*Sus scrofa*; Accession ACB87600.1), rabbit (*Oryctolagus cuniculus*; Accession XP_017195918), Egyptian fruit bat (*Rousettus aegyptiacus*; XP_015999857.1), cow (*Bos taurus*; NP_776791.1), and hedgehog (*Erinaceus europaeus*; XP_007525810.2) were comprised of their species mature ISG15 sequence identified by sequence homology, codon optimized, and had additional amino acid sequence GTEPGGRSGHHHHHH added to the C-terminal end. These constructs were placed into a pET-15b plasmid using the NdeI/BamHI restriction sites. The expression and purification of these proISG15s mirrored that of previously reported proISG15s [38]. In short, *E*. *coli* BL21 (DE3) cells containing these proISG15 constructs were grown in 6 L of LB broth containing 100 μg/mL ampicillin until an OD_600_ of 0.6 was reached. Expression was induced by the addition of IPTG to a final concentration of 0.5 mM then the culture was grown overnight at 18°C. Subsequently, bacterial cells were isolated via centrifugation at 6,000 x *g* for 10 min and stored at -80°C until purification. For purification, the cell pellets containing these ISG15s were suspended in Buffer A [500 mM NaCl, 50 mM Tris (pH 7.0), 1 mM Tris (2-carbozyethyl) phosphine hydrochloride (TCEP-HCl)]. The addition of 5 mg of chicken lysozyme per 500 mL of Buffer A was used to initiate lysis for 30 minutes at 4°C then sonicated on ice at 50% power with 5-second pulse increments for 6 minutes. The insoluble cell debris was separated via centrifugation for 30 min at 48,000 x *g*. The resulting supernatant was filtered with a 0.80 μm filter prior to flowing it over a high-density nickel agarose beads (GoldBio) equilibrated with Buffer A. The column was then washed using Buffer A supplemented with 30 mM imidazole prior to eluting the protein of the column using Buffer A supplemented with 300 mM imidazole. The eluted protein was further purified using a Superdex-S75 column equilibrated with Buffer B [200 mM NaCl, 50 mM HEPES (pH 7.0), 2 mM DTT]. These purified pro-ISG15s were concentrated to ~1–4 mg/mL for storage at -80°C until use. All OTU and ISG15 protein concentrations were determined through UV-visible spectroscopy at 280 nm using molar extinction coefficients experimentally derived by the method of Gill and von Hippel [58].

To generate the propargylamine derivatized sheep ISG15 and its C-terminal variant containing amino acids 79–156, sequence with codon for last glycine removed was inserted into pTYB2 using the NdeI/SmaI restrictions sites. As previously described [59], subsequent mutation was performed to alter the SmaI site to create the desired the RLRG sequence at the C-terminus of the two ISG15 constructs. These constructs were expressed and initially purified in the same way as the other ISG15 constructs. For purification, the only difference in the initial steps were the use of buffer C [75 mM NaCl, 50 mM sodium acetate, and 25 mM HEPES (pH 6.8)] augmented with 0.16% Triton X-100. Once the clarified supernatant was obtained, it was flowed over a chitin resin column pre-equilibrated with buffer C. The chitin resin was subsequently washed with 2 column volumes of buffer C and resuspended in 50 ml of buffer C supplemented with 260 mM sodium 2-mercaptoethanesulfonate (MESNA). The solution was then rotated gently overnight 4°C, and the chitin beads were recollected by gravity flow. The volume of the solution containing the thioester forms of sheep ISG15 and C-sheep ISG15 was reduced to 10 mL. To generate the final derivatized product of sheep ISG15-PA, or C-sheep ISG15-PA, 0.92 g of propargylamine and 240 μL of 5 M NaOH were added to the ISG15 thioester containing solutions and left to incubate overnight at 4°C.

### Protease activity assay with proISG15 substrates

Activity assays of OTUs originating from CCHFV, DUGV, ERVEV, NSDV, GANV, TAGV, QYBV, FARV, HpTV-1, ISKV, LPHV, DGKV, HAZV, and KUPEV with purified proISG15 derived from Egyptian fruit bat, northern tree shrew, rabbit, sheep, cow, fish, mouse, hedgehog, camel, vesper bat, and human were adapted from the previously reported methods [38]. Briefly, 20 nM OTU was tested for the ability to cleave 10 μM of each proISG15. Timepoints were taken over the course if an hour and the reactions quenched in 2x Laemmli buffer and boiling at 98°C for 5 minutes. Samples were run on BioRad Mini-PROTEAN^®^ TGX^^™^^ (OTU mutants with proISG15) or Mini-PROTEAN^®^ TGX Stain-Free^^™^^ pre-cast gels (wildtype proISG15 assays and mutant proISG15 assays). All the OTU mutant assays with proISG15 and assays involving fish proISG15 were visualized by Coomassie staining. Visualization of the remaining assay timepoints relied on Stain-Free technology that enhances the fluorescence of endogenous tryptophan. The gels were UV-activated for two minutes and subsequently imaged in a BioRad ChemiDoc^^™^^ Imaging system according to the manufacturer’s recommendations.

### Cellular deISGylase assay

OTU activity towards cellular conjugated ISG15 was determined using purified OTUs and cell lysates. Cell lysates containing conjugated human ISG15 were obtained by treating Huh7 cells with 1000 IU of IFN-β for two days before harvesting in triton lysis buffer (1% triton X-100, 20 mM Tris-HCl, 2.5 mM MgCl_2_). Alternatively, lysates were obtained by transfecting HEK293T cells with plasmids expressing V5-tagged sheep or human ISG15 (Genscript), and human Ube1L, UbecH8, and HERC5 using Trans-IT-LT1 (Mirus). Lysates were clarified by centrifugation (20 min at 13,000 rpm) and incubated for 20 min at room temperature with purified OTU. Samples were mixed with 4x Laemmli buffer and boiled at 95°C for 8 minutes. Proteins were separated on 4–12% Bis-Tris SDS-PAGE gels and transferred to nitrocellulose membranes using a trans-blot system (BioRad). ISG15 was detected using an ISG15 antibody (Proteintech # 15981-1-AP) or a V5 tag antibody (ThermoFisher Scientific #R960CUS). Tubulin was used as loading control marker (T5169, Sigma). Primary antibodies were detected with SuperSignal West Dura Fast Western blot kits (Thermo Fisher). Protein bands were visualized using a ChemiDoc MP system (BioRad).

### KUPEV OTU-C-sheep ISG15 and GANV OTU-sheep ISG15 complex formation

The procedure to form the KUPEV OTU-C-sheep ISG15 and GANV OTU-sheep ISG15 complexes was adapted from previously described methods [38]. In short, purified KUPEV OTU and GANV OTU was added directly to the C-sheep ISG15-PA and sheep ISG15-PA mixtures respectively in a 1:4 volume ratio. The solutions were then dialyzed in buffer D [250 mM NaCl, 25 mM HEPES (pH 7.0)] and buffer E [100 mM NaCl, 50 mM Tris (pH 8.0)] respectively overnight at 4°C. The dialyzed complex solution was then flowed through high-density nickel agarose beads pre-equilibrated with buffer D and buffer E respectively. The KUPEV OTU-C-sheep ISG15 complex was washed with buffer D supplemented with 30 mM imidazole and eluted with buffer D supplemented with 300 mM imidazole. The GANV OTU-sheep ISG15 complex was washed with buffer E supplemented with 10 mM imidazole and eluted with 300 mM imidazole. To further purify the GANV complex, the solution, dialyzed in 50 mM Tris (pH 8.0), underwent anion exchange chromatography, eluting from a MonoQ 10/100 column using a linear gradient from 0 to 1 M NaCl with 50 mM Tris (pH 8.0). To further purify the complexes, size exclusion was performed on a Superdex 75 column pre-equilibrated with buffer F [100 mM NaCl, 5 mM HEPES (pH 7.0), and 5 mM DTT] for KUPEV OTU-C-sheep ISG15 and buffer G [100 mM NaCl, 5 mM HEPES (pH 7.5), and 10 mM DTT] for GANV OTU-sheep ISG15. The KUPEV OTU-C-sheep ISG15 and GANV OTU-sheep ISG15 complexes were then concentrated to 12 mg/ml and 12.5 mg/ml respectively.

### Crystallization of KUPEV OTU-C-sheep ISG15 and GANV OTU-sheep ISG15

The KUPEV OTU-C-sheep ISG15 and GANV OTU-sheep ISG15 complexes were screened against a series of Qiagen NeXtal suites by hanging drop using a TTP Labtech Mosquito (TTP Labtech, Herfordshire, United Kingdom). For the KUPEV OTU-C-sheep ISG15 complex, the initial screens yielded small, cube-shaped crystals from a condition consisting of 0.1 M sodium acetate (pH 4.6) and 2.0 M potassium acetate. This condition was optimized using a follow up screen, varying concentrations from 2.1 M to 2.6 M potassium acetate and varying pH from 3.6 to 5.1. The final optimized crystals were grown in hanging drops with 2 ul of protein complex solution mixed 2:1 with mother liquor containing 0.1 M sodium acetate (pH 4.1) and 2.2 M potassium acetate. The crystals were flash cooled in a cryoprotective solution containing 0.1 M sodium acetate (pH 4.1), 3 M potassium acetate, and 30% glycerol. For the GANV OTU-sheep ISG15 complex, the initial screens yielded flat, hexagonal crystals from a condition containing 0.1 MES (pH 6.5) and 15% PEG 20,000. This condition was optimized using a follow up screen, varying concentrations from 8% to 19% PEG 20,000. The final optimized crystals were grown in hanging drops with 1 ul of protein complex solution mixed 1:1 with mother liquor containing 0.1 MES (pH 5.5) and 15% PEG 20,000. The crystals were flash cooled in a cryoprotective solution containing 15% PEG 20,000 and an 18% solution consisting of ethylene glycol, DMSO, and glycerol present in a 1:1:1 ratio (EDG).

All crystals were mounted under a dry N_2_ steam at 100 K. A data set for KUPEV OTU-C-sheep ISG15 was collected at the National Synchrotron Light Source II (Brookhaven National Laboratory, Upton, NY) on Life Science Biomedical Technology Research AMX beamline 17-ID-1 using a Eiger9M detector. Data were collected using wavelength 1 Å. Similarly, a data set for GANV OTU-sheep ISG15 was collected at the Advanced Photon Source (Argonne National Labs, Argonne, IL) on SBC-CAT beamline ID-18 using a Pilatus3 X 6M detector. Data were collected using wavelength 1 Å.

### Data processing and structure solutions

All X-ray images were indexed, strategized, integrated, and scaled using HKL2000 [60]. To create a cross-validation set from a random 5% of the reflections to be used throughout refinement, the CCP4 software suite was employed [61]. The initial phase solutions for the structures of KUPEV OTU-C-sheep ISG15 and GANV OTU-sheep ISG15 were obtained using molecular replacement via Phaser [62]. The search models for both KUPEV OTU-C-sheep ISG15 and GANV OTU-sheep ISG15 were homology models created by MODELLER [63] using the structures of CCHFV OTU-human ISG15 (PDB entries 3PHX and 3PSE) [43, 46], DUGV OTU-Ub (PDB entry 4HXD) [44], and ERVEV OTU-mouse ISG15 (PDB entry 5JZE) [38] as templates. The structures were refined initially using Autobuild [64] then iterative cycles of model building with Coot [65] and refinement with Phenix [66]. The Find Water COOT program function was used to initially add water molecules to 2Fo—F_c_ density peaks greater than 1σ and subsequently were assessed individually [67]. Molprobity was used to examine the final model of each structure to confirm the quality of the structures. The data collection and refinement statistics for each structure along are listed in Table 1. KUPEV OTU-C-sheep ISG15 (PDB entry 6OAR) and GANV OTU-sheep ISG15 (PDB entry 6OAT) have been deposited in the protein data bank.

### Mutant generation and enzymatic assays

Mutants of proISG15 and nairovirus OTUs were generated by the QuikChange approach using the manufacturer’s protocol (Agilent Technologies, Inc). The resulting PCR product was transformed into NEB-5α cells by heat shock (New England Biolabs), followed by plasmid purification and confirmation of mutants by sequencing. Confirmed mutant plasmids were transformed into BL21(DE3) or T7 Express cells by heat shock (New England Biolabs). The proISG15 cleavage assays were run as described above. Assays with Ub- and human ISG15-AMC for the OTU mutants were run in duplicate as previously described [38, 41, 44]. Confirmation of Ub-AMC activity for HAZV, TAGV, FARV, DGKV, HpTV-1, LPHV, QYBV, and ISKV were run with an adapted protocol (S2C Fig). Assays were run in triplicate with 4 nM OTU against 1 μM Ub-AMC in a 30 μL reaction volume.

### Accession numbers

Final protein structures were deposited in the Protein Data Bank with IDs for 6OAR and 6OAT for KUPEV OTU–C-sheep ISG15 and GANV OTU–sheep ISG15 complexes respectively.

## Supporting information

S1 Fig Expanded nairovirus OTU sequence alignment. Sequence alignment of the OTUs from the fourteen viruses included in this study. Annotated as in Fig 1A.
(TIF)

S2 Fig Related to Fig 2. (A) OTU-proISG15 cleavage assays for HAZV, TAGV, FARV, DGKV, HpTV-1, LPHV, QYBV, and ISKV. Data obtained as described in Fig 2 and the Materials and Methods. (B) Western Blots of OTU activity against ISGylated substrates in cellular lysates. (C) Reference Ub-AMC activity for OTUs. DGKV, HpTV-1, and LPHV are known to have poor/negligible DUB activity [44]. Values are the mean ± standard deviation of three independent experiments.
(TIF)

S3 Fig Related to Fig 3. (A) Surface rendering of the KUPEV OTU with the major regions forming the interface indicated. (B) Electrostatic interactions between KUPEV OTU (green) and sheep ISG15 (purple) in a region peripheral to the main interface.
(TIF)

S4 Fig USP18 sequence alignment. Sequence alignment of USP18 from human (Accession: CAG33497.1), mouse (Accession: CAJ18436.1), cow (Accession: XP_005887504.1), pig (Accession: NP_998991.1), hedgehog (Accession: XP_016048336.1), Egyptian fruit bat (Accession: XP_015980899.1), rabbit (Accession: XP_017193977.1), and camel (Accession: P_010992102.1). The catalytic triad is shown in black boxes. Regions forming the interface with ISG15 are noted by blue bars based on a mouse USP18-ISG15 X-ray crystal structure (PDB entry 5CHV).
(TIF)

S1 File Validation report for KUPEV OTU-C-sheep ISG15. (PDF)

S2 File Validation report for GANV OTU-sheep ISG15. (PDF)
