# Supplementary figures and images for: Determining the molecular drivers of species-specific interferon-stimulated gene product 15 interactions with nairovirus ovarian tumor domain proteases
Source: PLoS One. 2019 Dec 23;14(12):e0226415. doi: 10.1371/journal.pone.0226415 (PMC6927636; doi:10.1371/journal.pone.0226415)

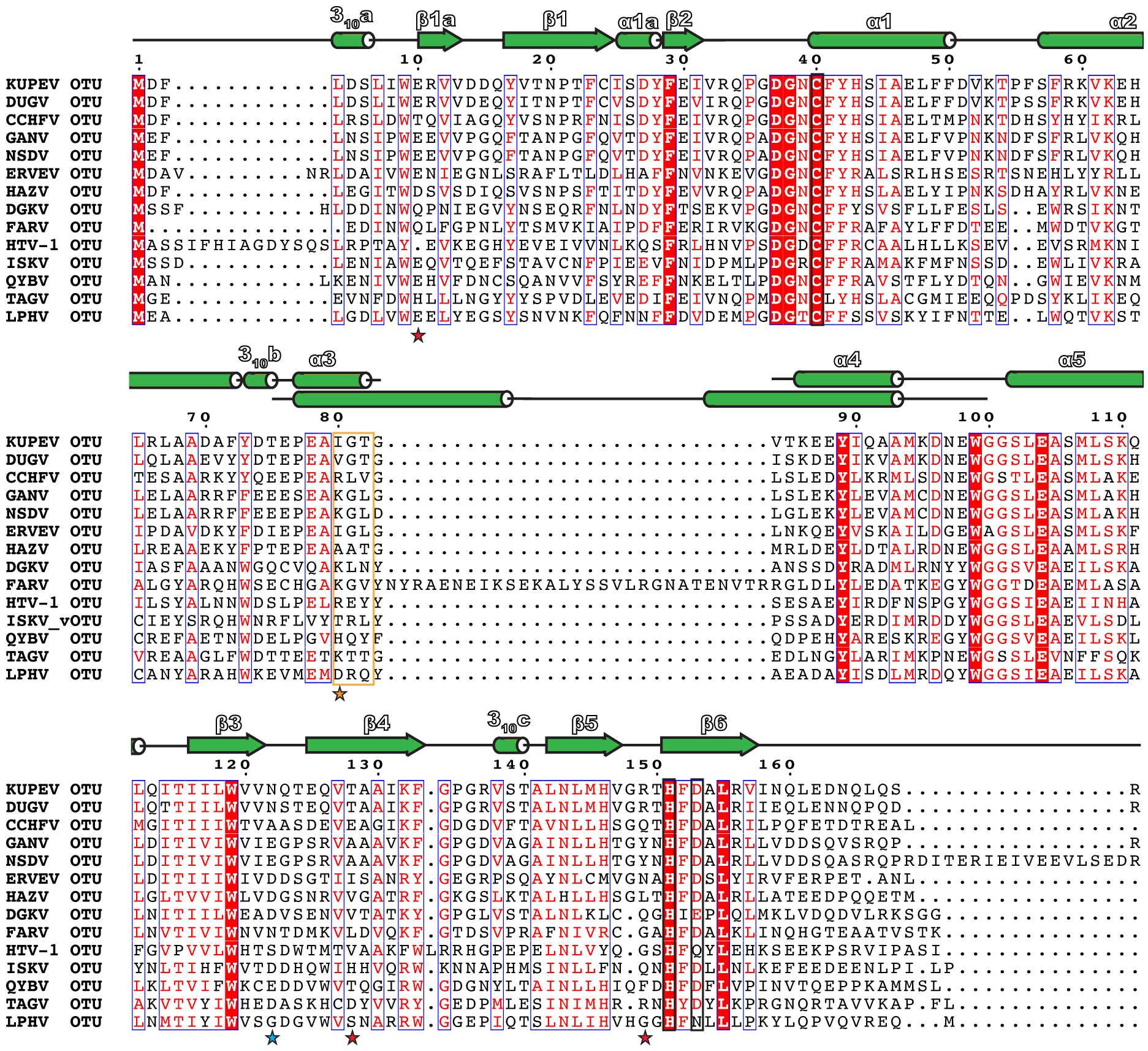

Supplement: S1 Fig — Sequence alignment of the OTUs from the fourteen viruses included in this study. Annotated as in Fig 1A. (TIF) [file pone.0226415.s001.tif]

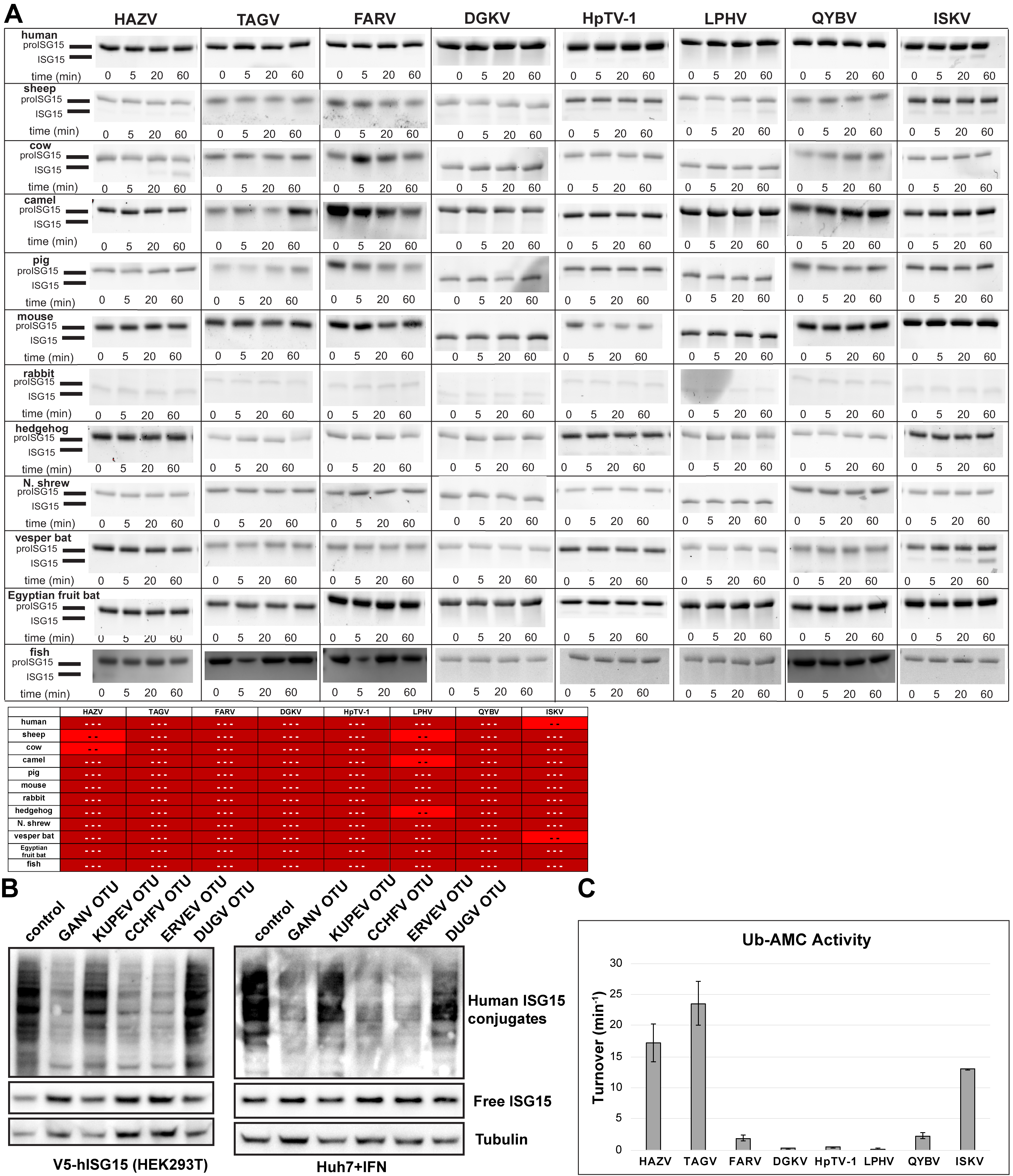

Supplement: S2 Fig — (A) OTU-proISG15 cleavage assays for HAZV, TAGV, FARV, DGKV, HpTV-1, LPHV, QYBV, and ISKV. Data obtained as described in Fig 2 and the Materials and Methods. (B) Western Blots of OTU activity against ISGylated substrates in cellular lysates. (C) Reference Ub-AMC activity for OTUs. DGKV, HpTV-1, and LPHV are known to have poor/negligible DUB activity [44]. Values are the mean ± standard deviation of three independent experiments. (TIF) [file pone.0226415.s002.tif]

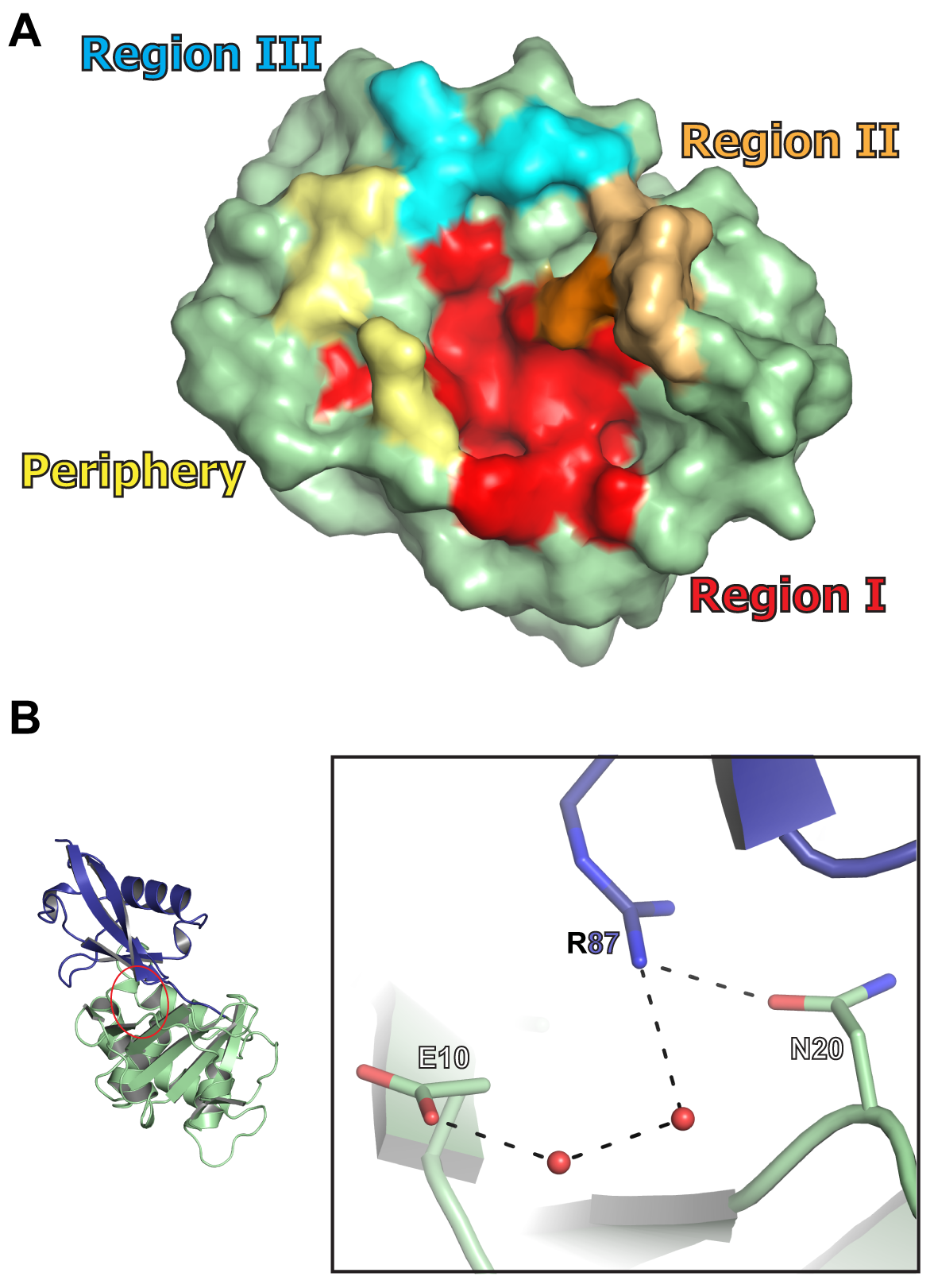

Supplement: S3 Fig — (A) Surface rendering of the KUPEV OTU with the major regions forming the interface indicated. (B) Electrostatic interactions between KUPEV OTU (green) and sheep ISG15 (purple) in a region peripheral to the main interface. (TIF) [file pone.0226415.s003.tif]

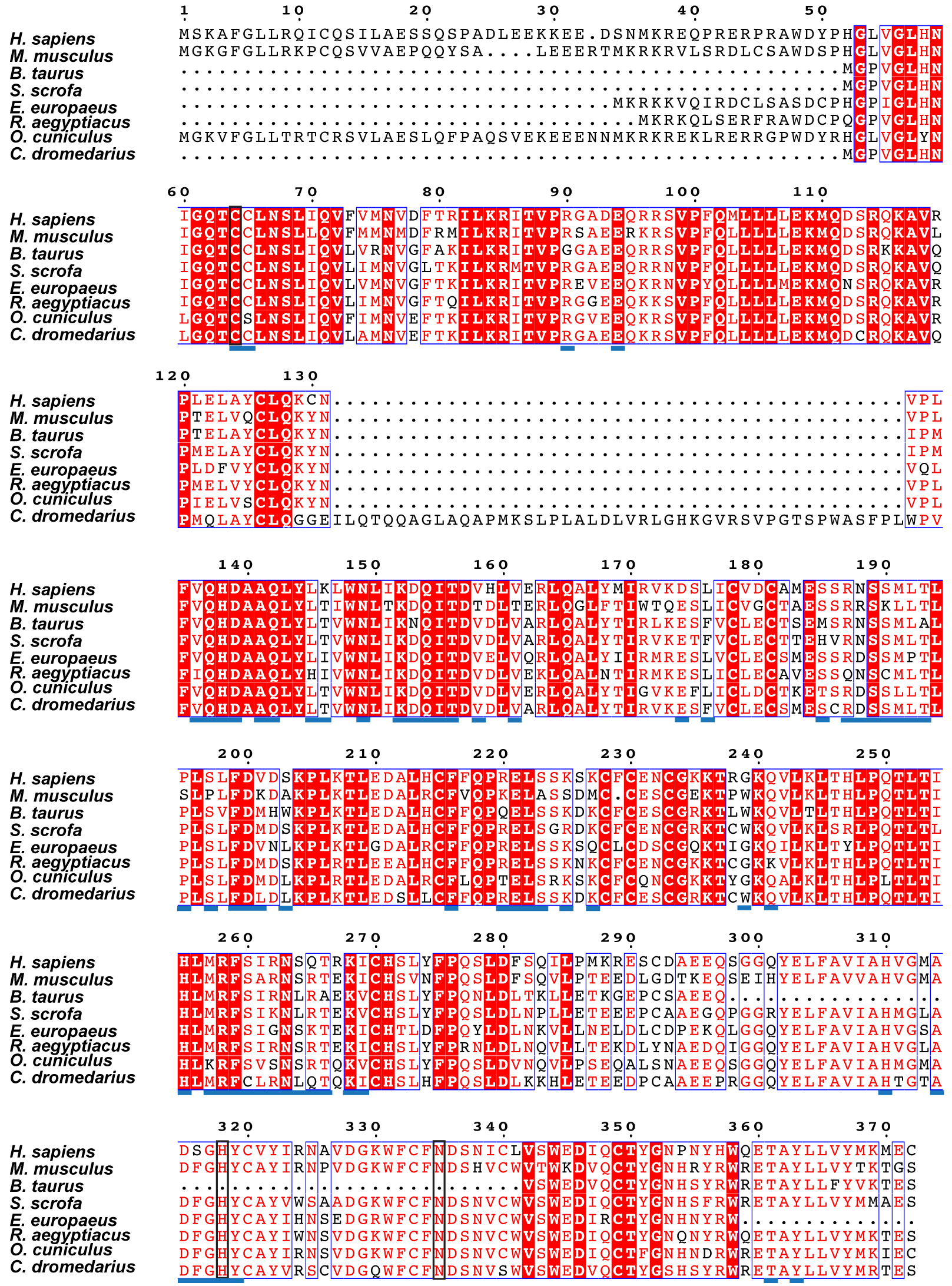

Supplement: S4 Fig — Sequence alignment of USP18 from human (Accession: CAG33497.1), mouse (Accession: CAJ18436.1), cow (Accession: XP_005887504.1), pig (Accession: NP_998991.1), hedgehog (Accession: XP_016048336.1), Egyptian fruit bat (Accession: XP_015980899.1), rabbit (Accession: XP_017193977.1), and camel (Accession: P_010992102.1). The catalytic triad is shown in black boxes. Regions forming the interface with ISG15 are noted by blue bars based on a mouse USP18-ISG15 X-ray crystal structure (PDB entry 5CHV). (TIF) [file pone.0226415.s004.tif]
